# Mapping the Elephants of the 19th Century East African Ivory Trade with a Multi-Isotope Approach

**DOI:** 10.1371/journal.pone.0163606

**Published:** 2016-10-19

**Authors:** Ashley N. Coutu, Julia Lee-Thorp, Matthew J. Collins, Paul J. Lane

**Affiliations:** 1 BioArCh, Department of Archaeology, University of York, York, United Kingdom; 2 Department of Archaeology, University of Cape Town, Rondebosch, South Africa; 3 Research Laboratory for Archaeology, University of Oxford, Oxford, United Kingdom; 4 Department of Archaeology and Ancient History, Uppsala University, Uppsala, Sweden; 5 School of Geography, Archaeology & Environmental Science, University of the Witwatersrand, South Africa; Museum für Naturkunde, GERMANY

## Abstract

East African elephants have been hunted for their ivory for millennia but the nineteenth century witnessed strongly escalating demand from Europe and North America. It has been suggested that one consequence was that by the 1880s elephant herds along the coast had become scarce, and to meet demand, trade caravans trekked farther into interior regions of East Africa, extending the extraction frontier. The steady decimation of elephant populations coupled with the extension of trade networks have also been claimed to have triggered significant ecological and socio-economic changes that left lasting legacies across the region. To explore the feasibility of using an isotopic approach to uncover a ‘moving frontier’ of elephant extraction, we constructed a baseline isotope data set (δ^13^C, δ^15^N, δ^18^O and ^87^Sr/^86^Sr) for historic East African elephants known to have come from three distinct regions (coastal, Rift Valley, and inland Lakes). Using the isotope results with other climate data and geographical mapping tools, it was possible to characterise elephants from different habitats across the region. This baseline data set was then used to provenance elephant ivory of unknown geographical provenance that was exported from East Africa during the late nineteenth and early twentieth centuries to determine its likely origin. This produced a better understanding of historic elephant geography in the region, and the data have the potential to be used to provenance older archaeological ivories, and to inform contemporary elephant conservation strategies.

## Introduction

Eastern and South-Eastern Africa are known to have been major sources of elephant ivory supplying the Mediterranean world, Western Europe, the Persian Gulf, India and China for at least the last two millennia [1–5]. The geographical origins of this exported ivory undoubtedly shifted over time, but information is largely lacking on precisely which locales were the primary suppliers during particular centuries and why these shifts occurred. The scale of extraction also changed over time, with the limited documentary sources suggesting a steady increase in the trade with India and China from ca. AD 1500 and perhaps earlier [6–8]. The colour, texture, and working properties of East African elephant ivory made it particularly desirable, and demand escalated in Europe and North America during the nineteenth century [9] encouraged by the industrialisation of ivory working and processing industries for the manufacture of cutlery-handles, piano-keys, billiard balls and other diverse household objects [10–12]. The growth in demand for such ivory products fuelled, and was fuelled by, wider changes in the aesthetics of taste, social distinction and patterns of conspicuous consumption among a growing middle class in both Europe and North America [13,14]. It was in part also shaped by the desires of East African consumers for the imported commodities used by caravan traders to acquire ivory [15]. Among the other factors that contributed to the greater availability of East African ivory in global markets were the pre-existing Indian trade networks [16], the development of a mercantile economy on Zanzibar following relocation of the Omani court to Zanzibar in the late 1830s [7], and the entry of American vessels, especially from Salem, Massachusetts, into the Indian Ocean trading system at around the same time [17]. By 1891, 75% of the entire world’s supply of ivory was shipped from Zanzibar [9,18], with estimates of East African exports ranging from 8,000 to 30,000 tusks per year for the latter half of the nineteenth century [9,19,20].

These estimates, which speak to the scale of ivory extraction in East Africa, are primarily based on nineteenth century trade records of ivory exports, principally from Zanzibar [7,9]. However, aside from patchy observations concerning elephant distributions made by early European explorers and missionaries [20,21], little is known about either the precise geographical origin of the ivory, whether changes in the location of preferred extraction areas occurred, or whether elephants were locally hunted to extinction. It is important to know each of these for at least three reasons. First, elephants are major ecological architects, and their local extirpation can result in significant habitat change stimulating regrowth of bushy vegetation and secondary woodland, as documented in Tsavo (SE Kenya) in the mid-twentieth century [22]. The presence of large herds of elephants in the landscape also has a range of other consequences for regional vegetation patterns and biodiversity more generally [8,23,24]. Secondly, sustained, large-scale ivory extraction likely had significant impacts on elephant reproduction patterns [25,26] and genetic diversity [27]. Finally, the expansion of the ivory trade is believed to have triggered significant socio-ecological and political change [28] as communities along the trade routes and in ivory extraction areas diversified their economic strategies and labour relations to take advantage of the trade opportunities. This resulted in the emergence of specialist hunters, porters and middlemen [29–31], and the founding of new settlements (such as Ujiji on Lake Tanganyika), several of which became prosperous trading hubs [32–34]. It is impossible to understand these impacts, however, without knowing where the ivory was extracted at different times, which elephant populations were most affected, and therefore which habitats likely changed.

**Fig 1:**
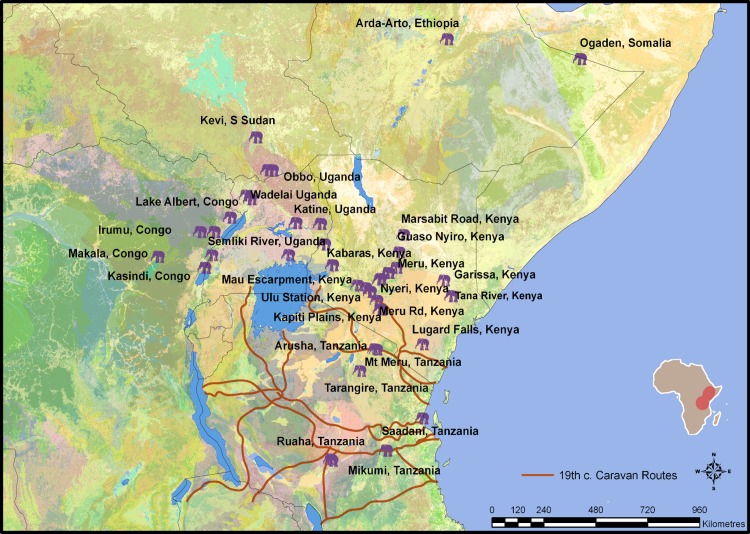
Location of historic and modern provenanced East African elephant tissue samples. Caravan routes adapted from [36] and base maps of global vegetation/land surface cover from [37] European Space Agency 2010 & UCLouvain GlobCover Project and geological map provided by the French Geological Survey (BRGM) through SIGAfrique of bedrock age at a scale of 10 metres with the permission of OneGeology [38].

From the available historical sources it is known that in the early part of the nineteenth century, most ivory was brought to the coast by groups residing farther inland [31], although trade caravans were already exploiting coastal elephant populations (e.g. [2]). By the mid-nineteenth century, however, trade caravans were being organised almost entirely from the coast and coastal traders were expanding their networks inland throughout Tanzania and northern Kenya (Fig 1; [35]). Drawing on these records, Sheriff [7] suggested that the ivory trade in the nineteenth century was a moving frontier, such that elephant herds living near the coast were the first to be intensively exploited from ca. 1830 onwards, as they were most readily accessible to trading expeditions from coastal ports. Others such as Thorbahn [8] and Håkansson [20] argue that large areas of the interior of East Africa were exploited for ivory prior to the nineteenth century trade boom, as the global demand for ivory was already substantial, making it likely that areas other than the coastal hinterland were already being exploited [8,20]. To better grasp the spatial distribution of ivory extraction across eastern Africa, alternative sources of information need to be explored. In line with this, we report here the first use of the isotopes of carbon, nitrogen, oxygen, and strontium, in combination, to source historic ivory traded from East Africa. Using this baseline data set, we explore the potential of using isotope data to geographically provenance historic ivory to specific areas of the region in order to understand historic extraction patterns. The unprovenanced data set we use to test the baseline data derives from collections that post-date 1890, but the baseline data have the potential to be used for sourcing ivory traded in earlier periods of the 19^th^ century.

## Sourcing traded ivory using isotope analysis

Isotope analysis has been applied to understand current distributions of elephants across Africa [39–41], as well as historic diet patterns [42,43] and contemporary feeding ecology [44–46]. Most recently, a multi-isotope approach was evaluated for forensic wildlife purposes to trace ivory across sub-Saharan Africa [47]. The research reported here uses isotope analysis to track the historic trade that changed the distribution of elephants on the landscape. Isotope ratios preserved in the tissues of elephants reveal several aspects of the ecological history of the animal, including its diet and habitat. Thus, each isotope analysis conducted for this research (^13^C/^12^C, ^15^N/^14^N, ^18^O/^16^O and ^87^Sr/^86^Sr) informs about where a historic elephant roamed within East Africa, given the principles of isotope ecology in elephants outlined below.

In African habitats, most grasses utilise the C_4_ pathway of plant photosynthesis, while trees, shrubs, forbs, and other bushy vegetation utilise the C_3_ pathway. The two photosynthetic pathways discriminate differently between the carbon isotopes with the result that the two groups have distinct δ^13^C values (the delta symbol (δ) indicates that the value is a measured ratio of ^13^C/^12^C relative to a standard reported in parts per thousand (‰)) [48,49]. C_3_ plants in tropical forests have significantly lower δ^13^C values still, due to the combined effects of low light, and effects of CO_2_ recycling beneath the forest canopy [50–52]. Elephants are mixed feeders, relying on a wide variety of plant species from trees to tropical grasses [53–57], and they inhabit a wide range of habitats, from forests to savannas. Since elephants eat both C_3_ and C_4_ vegetation at least partly according to their presence in the habitat, the δ^13^C value measured in the elephant’s tissue also reflects the vegetation available in the habitat [56]. The ivory collagen δ^13^C values of forest elephants (*Loxodonta cyclotis*), living mainly in the closed tropical forests of central and western Africa, are significantly more negative than those of elephant feeding exclusively on C_3_ biomass in more open habitats [39, 56]. Savanna or bush elephants (*Loxodonta africana*), living mainly in eastern and southern Africa savanna, have a mixed diet of C_3_ and C_4_ vegetation with collagen δ^13^C values between 21‰ and -12‰ [42,43,45,57]. We expect to see similar separations in the δ^13^C values of our data set based on density of tree cover in the habitat, as elephant habitats in the interior Lakes region of East Africa were dominated by closed-canopy forest, whereas coastal and Rift elephant habitats included mosaics of tree, bush and grassland (Fig 1).

The variability of stable nitrogen isotopes (^15^N/^14^N) in ecosystems reflects the balance between biologically available nitrogen, fixation, and complex recycling and re-release of N_2_ within the biosphere [58]. Atmospheric N_2_ is globally uniform in isotope composition, with a low δ^15^N composition (0‰ by definition). On land, soils and plants tend to be slightly ^15^N-enriched compared to atmospheric N_2_ [59] so their δ^15^N values are typically about 1–4‰, subject to variability related to environmental aridity, leaching (with high precipitation), anoxia and salinity [60–62]. Nitrogen isotope ratios in elephant tissue largely reflect the composition of the vegetation they consume, which is influenced by nitrogen recycling in the soil in which the vegetation grows, and the plants and plant part they consume [61,63–65]. In most humid rainforest habitats with organic-rich soils, plant δ^15^N values tend to converge around 5–6‰ (mean value for plants in Kibale Forest, Uganda and Amazon Forest, Brazil) [66–68]. In open, arid environments ^15^N-enrichment can occur due to the loss of volatile nitrogen in the soil, leading to higher δ^15^N values in plants and animals [69,70]. Although it has been suggested that soil and plant δ^15^N is inversely related to rainfall [62], in practice this relationship is only seen in locations where mean annual rainfall is less than 400 mm [61,71], and it is highly variable. For elephants living in arid environments (Ethiopia, the Namib, Somalia), δ^15^N values of up to 12–13‰ have been observed [39,72]. We expect to see a similar trend of high δ^15^N values for arid environments and lower, yet variable values in elephants from rain forest and mosaic habitats in eastern Africa.

The sources of oxygen for an elephant include atmospheric oxygen, drinking water, leaf water [73], and oxygen from carbohydrates in plants. Atmospheric oxygen can be discounted since it is well-mixed, leaving the primary external influences on ^18^O/^16^O variability as the isotopic composition of local rainfall [74], moderated by the influence of evaporation. The isotopic composition of drinking water is dependent on local climatic and geographical factors [73,75,76], including distance from the coast, altitude and latitude due to rainout effects in the former case and cooler air temperatures in the latter two [77]. Areas farther from the source of moisture (the oceans) and at higher altitudes generally have lower δ^18^O values in water, and also consequently in plant tissues [78,79]. Plants respond to moisture availability in their habitat so leaf water and plant sugars are important sources of variability in mammal tissues. Plants that grow in cooler, wetter, or shaded habitats exhibit lower evaporation rates from foliage [80,81], while those in more open, arid habitats experience higher evapotranspiration, with the result that the latter are generally enriched in ^18^O [82,83]. Thus, we expect East African elephants living in more open, arid environments (such as Tana River, Kenya) to have higher δ^18^O values, and those living in more humid, closed canopy forests and high altitude habitats (such as the Mau Escarpment/Mount Kenya, Kenya, and Mount Meru, Tanzania) to have lower δ^18^O values.

The ^87^Sr/^86^Sr incorporated into elephant tissue reflects, to a large degree, the strontium of the bedrock on which elephants roamed. Bedrock strontium isotope composition reflects the age of the geology and the rubidium content. However the strontium that an animal incorporates into its tissues does not directly reflect the bedrock geology, but rather the strontium that is made available in soils to plants and then to animals. This ‘bioavailable’ strontium is affected by the amount of weathering that occurs from bedrock to soil. General patterns of higher or lower ^87^Sr/^86^Sr values do exist when compared to the type and age of the bedrock geology, especially in such a diverse and widespread area as East Africa. Tissue ^87^Sr/^86^Sr values represent an average of the range of values from bedrock over which each elephant roamed during the time of tissue formation. The geology of East Africa is highly variable (See Fig 1) and ranges from young volcanics in the Rift Valley to much older basement found in the Congo Basin. Elephants which roamed on Rift Valley basalts (e.g. Arusha, Tanzania) are expected to have the lowest ^87^Sr/^86^Sr values in the region, (0.7030 to 0.7045; [84]). Along the East African coast (e.g. Saadani/Mikumi, Tanzania), ^87^Sr/^86^Sr values are expected to reflect a marine-like quaternary sedimentary range, strongly influenced by coastal, windblown sands and the breakdown of coral in the soil (~0.710). The highest values are expected in the regions with the oldest geology where rubidium content is also high, such as Precambrian granites (e.g. Ruaha, Tanzania) found farther into the interior and in the Great Lakes region (> 0.710; [85]).

## Material and Methods

### Sample Selection

Modern and historic baseline elephant specimens from known habitats across East African ecological zones were sampled from museum collections in Africa, US, UK and Europe (Fig 1, S1 Table) and analysed for the isotopes of carbon (δ^13^C), nitrogen (δ^15^N), oxygen (δ^18^O), and strontium (^87^Sr/^86^Sr). Most samples were derived from the Powell-Cotton Museum (near Margate, Kent, UK) natural history collection assembled by the big game hunter Major Powell-Cotton (b. 1866, d. 1940). The benefit of using this collection is the detail and quality of the accompanying archival evidence regarding his hunting expeditions which helped contextualise the isotope results for these samples [86]. Particular emphasis was placed on collecting samples from areas surrounding the known nineteenth century caravan routes, as this would likely be where traded elephant ivory was initially sourced.

Unprovenanced samples comprised piano keys, cutlery handles, and other ivory fragments from museum collections including the Hawley Collection, Sheffield (UK), House of Wonders, Zanzibar (Tanzania), Deep River and Ivoryton Museum, Connecticut (USA), and one archaeological site (Korogwe, Tanzania). These samples are known to have been traded within and from East Africa, from ca. 1890 to the mid-20^th^ century. Dating of these samples is based on archival and collection records, which typically indicate when the ivory was manufactured. Therefore, it is likely that the material found in European and American collections dating to the early and mid-20^th^ century in particular was traded out of East Africa as raw ivory prior to this, though it is difficult to know precisely when. Ivory was too valuable and sought after not to be used soon after its arrival in the warehouse for manufacture, but the dates for the samples are still approximate.

Due to the fragmentary nature of the collections and restrictions on invasive sampling of some museum material, it was not always possible to sample ivory directly. Consequently, bone and molar teeth were also analysed. Bone is a dynamic tissue that re-models, whereas molars do not–they are formed during a limited period of the animal’s life and do not undergo any changes after formation. Ivory is a modified upper incisor, or tusk, that grows incrementally throughout the lifetime of the elephant, and therefore archives a continuous record [43,87]. In order to minimise the effect of inhomogenous, incremental tissues and differences in turnover, we sampled larger areas of molars or the tusk, in order to average multiple growth increments, and sampled in the same area of the molar, bone, or tusk in each case. For example, the (most recently formed) proximal end of the tusk was sampled (elephants use the distal end in daily activities, so this is worn away throughout their lifetime). Where possible, multiple tissue samples were obtained from the same specimen, including tail hairs, to assess the variability of isotope values over short-term time scales and these data are reported separately [86,88]. These results suggest that intra-annual variability amongst forest elephants is lower than that found in savanna elephants, likely because of lower seasonal shifts in palatable vegetation and water availability, which is in accordance with other studies [43,45]. While analyses of different tissues from the same animal demonstrated that an individual elephant may well range across different ecological zones during its lifetime, large pieces of ivory, bone, and molar that are homogenised in the laboratory give averaged isotope values and relate to where an elephant spent the majority of its life over multiple seasons.

Another sampling-related issue is that most historic elephant tissues collected by big game hunters were from male elephants (See S1 Table), because they are solitary and their tusks tend to be larger and hence were more sought after. Males, specifically lone bulls, have larger home ranges than females due to the matriarchal structure of elephant family groups [89–91]. Females have higher nutritional demands and the social responsibility of feeding the juveniles in the matriarchal group, so they tend to be more conservative about staying close to reliable sources of water and food [89–91]. Thus, sex and vegetation heterogeneity in the habitat also influence the variability of individual elephant isotope values within a population.

### Carbon and Nitrogen

For collagen extraction, either whole chips or powder were collected using a clean diamond-tip drill bit on a Dremel hand drill. Museum samples from Europe as well as East African samples (CITES permit 451717, 318736, 22225) were measured at the University of Bradford (UK) stable light isotope laboratory, whilst samples from museums in the USA were measured at the University of Illinois Urbana-Champaign (USA) stable isotope laboratory due to CITES restrictions on their export. Collagen extraction in both labs followed an adapted method of gelatinization and filtration following the Longin method [92] and in Illinois followed the method by Ambrose [93]. Briefly, cleaned chips or powdered samples were demineralised in 0.5M HCl at a temperature of 4°C for a period of 1–5 days then rinsed with de-ionized water to neutrality [92]. After this, the samples were put in a 0.01M HCl (pH 3) solution and heated to 70°C for 48 hours to denature the collagen [92]. The modern and historic museum samples were then centrifuged and filtered to remove large debris (60–90 μm Ezee® filter) whereas the few archaeological samples also went through a second filtering (30,000 nm ultrafilters) to remove contaminants. All of the samples were then frozen and lyophilized for analysis.

In Bradford, samples were weighed into tin capsules for combustion into N_2_ and CO_2_ gases on a Thermo Flash Elemental Analyser 1112 attached to a Delta plus XL mass spectrometer for measurement of ^13^C/^12^C and ^15^N/^14^N ratios as well as elemental compositions (%C, %N). In Illinois, samples were similarly combusted on a Carlo Erba NC 2500 Elemental Analyser coupled to a Finnigan MAT 252 mass spectrometer. In both laboratories, samples were measured together with internal laboratory standards (fish gelatine, whale bone collagen) as well as international standards (e.g. ammonium sulfate (IAEA-N2), sucrose (IAEA-CH), and thiourea (Sigma-Aldrich) to ensure inter-lab reproducibility of results. The results listed in S1 Table are expressed in parts per thousand (‰) as delta (δ) relative to international standards of PeeDee Belemnite (VPDB) for carbon and Ambient Inhalable Reservoir (AIR) for nitrogen. Repeatability of the internal standards was less than 0.2‰ for δ^13^C and δ^15^N. All collagen samples fell within a normal atomic C:N range of between 2.8 and 3.6, used as standard quality control measure [93].

### Carbon and Oxygen

For analysis of bioapatite carbonate, pieces of bone/molar/ivory were either drilled using a small diamond tip on a Dremel hand drill or homogenized in a SpexMill. Purification followed protocols by Sponheimer [94], paying particular attention to the acetic acid step, which reacts extremely quickly with modern dentine (ivory and molar) samples. Briefly, a NaOCl solution (~1.7% v/v) was added to 10–20 mg of powder in a 2ml centrifuge tube for 3 hours to eliminate the organic content, then centrifuged and rinsed with de-ionized water several times. After this, a 0.1M acetic acid (CH_3_COOH) solution was added to the samples, reacted for 5 minutes, and then centrifuged and rinsed several times in de-ionized water. Samples were frozen and lyophilized. In Bradford, dried, powdered samples were weighed into glass tubes for reaction with 100% phosphoric acid at 70°C in a Thermo Gasbench II interfaced with a Delta V mass spectrometer. In Illinois, samples were analysed on a dual inlet Thermo Finnigan Kiel III device interfaced with a Finnigan MAT 252 mass spectrometer. All δ^18^O measurements are reported as compared to the Vienna mean standard ocean water (VSMOW) while δ^13^C values are expressed relative to the VPDB standard. International standards measured alongside the samples in both laboratories were NBS 18 and NBS 19. Analytical error was less than 0.2‰ for both isotopes.

The δ^13^C values of modern samples were adjusted for the fossil fuel effect in the atmosphere, using as starting year 1896 AD (the oldest sample in the data set). The correction factor is calculated from the fossil fuel curve for the year to which the sample dates according to museum and archival records [95].

### Strontium

Strontium analysis was carried out at the University of Illinois, Urbana-Champaign (USA) and the University of Cape Town (South Africa) for reasons related to CITES restrictions. An aliquot of the bone, ivory or molar dentine powdered as above for carbon and oxygen isotope analysis was utilised for strontium separation in both labs using chromatographic separation with Eichrom Sr spec resin, following protocols for solution MC-ICP-MS [96]. In both laboratories, the powdered sample was dissolved in nitric acid (3M HNO_3_), then loaded onto chromatographic separation columns with a slurry of Sr spec resin in the base of the column. Once the sample was loaded onto the column, a series of 3M HNO_3_ elutions removed other heavy elements allowing isolation of purified strontium. The purified strontium was then eluted with 0.05M HNO_3_ into a Teflon beaker, dried down at 90°C and reconstituted in 2ml of 0.2% HNO_3_ for introduction directly into the mass spectrometer for isotope analysis. A NuPlasma multi-collector inductively coupled mass spectrometer (MC-ICP-MS) was used for analysis in both laboratories. The international strontium standard NIST 987 was measured as a control, giving 0.710257 ±0.000057 (2σ) (reference value = 0.710255) based on multiple replicates (n = 57).

### Analysis Tools

**Fig 2:**
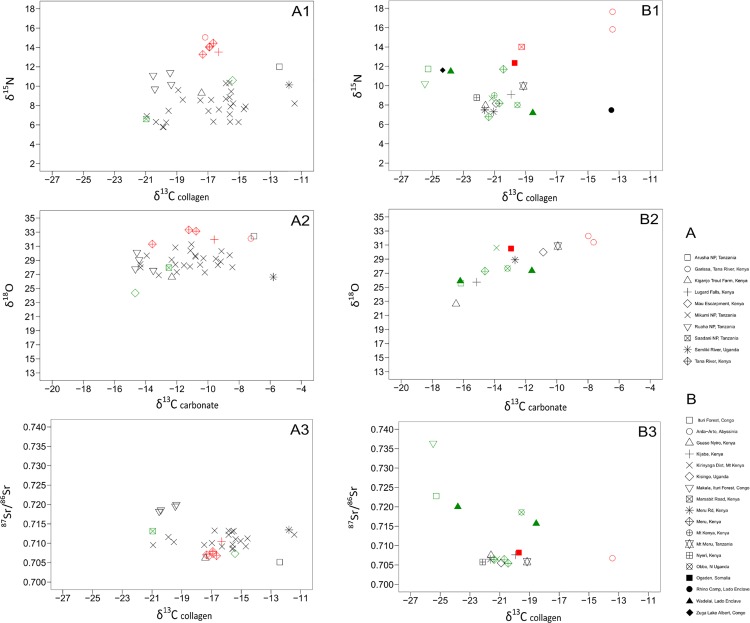
δ^13^C, δ^15^N, δ^18^O and ^87^Sr/^86^Sr values for modern and historic provenanced elephant samples from East Africa. (A1-3) Three plots of modern (post-1950) elephant tissue samples collected from museum specimens and national parks in Tanzania (see S1 Table). Colours of all samples correspond to habitat following [41] for East African elephant habitats: green is forest/mountain, black is savanna mosaic (incorporates a wide range of woodland/bushland/grassland habitats) and red is arid (incorporates arid bushland and grassland habitats), with habitat descriptions following [97]. (A1) δ^13^C and δ^15^N values of collagen, (A2) δ^13^C and δ^18^O values of carbonate, and (A3) δ^13^C and ^87^Sr/^86^Sr values, from collagen and carbonate respectively. (B1-3) Three plots of historic (1896–1909) elephant tissue samples collected from museum specimens (see S1 Table). (B1) δ^13^C and δ^15^N values of collagen, (B2) δ^13^C and δ^18^O values of carbonate, and (B3) δ^13^C and ^87^Sr/^86^Sr values, from collagen and carbonate respectively.

**Fig 3:**
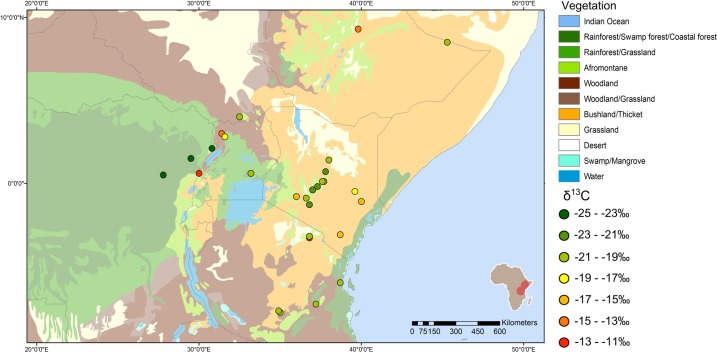
Map of δ^13^C values and vegetation. Circle colour represents the δ^13^C value of each sample from that location. The base map of vegetation cover with legend is a simplified version of [98].

**Fig 4:**
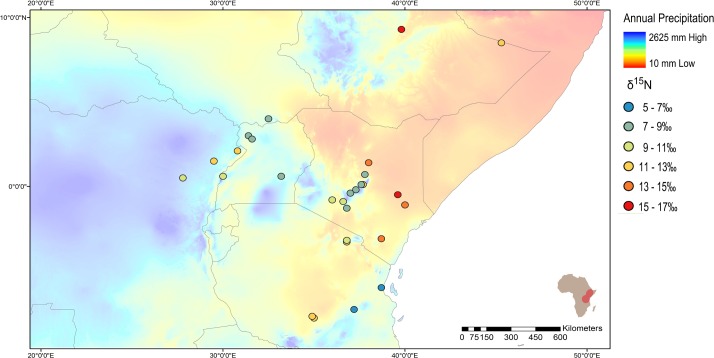
Map of δ^15^N values and annual precipitation. Circle colour represents the δ^15^N value of the sample from that location. Base map is of annual precipitation (mm) from the WorldClim database [101].

**Fig 5:**
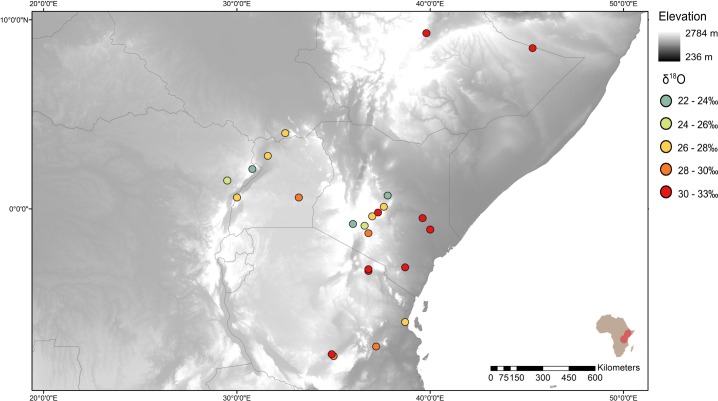
Map of δ^18^O values and elevation. Circle colour represents the δ^18^O value of the sample from that location. Base map is of elevation above sea level (m) from the WorldClim database [101].

**Fig 6:**
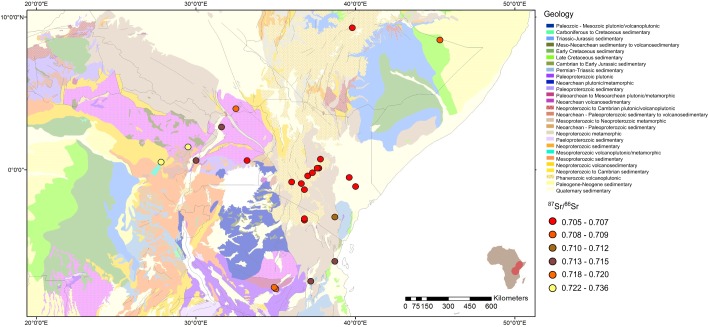
Map of ^87^Sr/^86^Sr values and geology. Circle colour represents the ^87^Sr/^86^Sr value of the sample from that location. Base map is of bedrock geology provided by the French Geological Survey (BRGM) through SIGAfrique of bedrock age at a scale of 10 metres with the permission of OneGeology [38].

Vegetation information was sourced from [97] and the digitised version from [98]. For scatterplots (e.g. Fig 2), the colours of the symbols are grouped according to their habitat of forest/mountain, savanna mosaic, and arid, adapted from [57] and based on the vegetation classifications in Fig 3 [97]. Historic climate data from the British Atmospheric Data Centre and Climate Research Unit time-series global grids are from 1901 [99,100]. Modern climate data (elevation and annual precipitation) in Figs 4 and 5 are from the World Clim Database [101]. The geology map in Fig 6 derives from the AfriCover database and OneGeology portal courtesy of the French Geological Survey (BGRM) [38]. Principle component analysis (PCA) was run in the R statistical software package [102].

## Results

The δ^13^C and δ^15^N results measured on the collagen of elephants from the modern data set range from -11.4 to -21.0‰ and 5.7 to 15.0‰, respectively, shown in Fig 2 (A1). The δ^13^C results show that most elephants were mixed feeders, consuming both C_3_ and C_4_ vegetation. Elephants from three locations (red symbols: Garissa, Lugard Falls, and Tana River) are separated from the main cluster of data points due to their high δ^15^N values (13 to 15‰). These high values likely reflect the aridity of these locations, which have highly seasonal, low annual rainfall–precipitation in the dry season is on average less than 50mm and in total less than 500mm per year [101].

Collagen δ^13^C and δ^15^N data for the historical ivory in Fig 2 (B1) fall into three clusters. The first (extreme left of the graph, δ^13^C = -23 to -25‰) consists of forest elephants that browsed in closed canopy forests dominated by C_3_ vegetation. This cluster includes the historic elephants that ranged in habitats farther into the East African interior and nearer the Great Lakes region, such as the Ituri Forest, Lake Albert, Lado Enclave Camp, and Makala samples. Their δ^13^C values are similar to those documented [41] for modern Central African elephants living in closed canopy forests (-24±1.3‰, n = 11). The second and largest cluster of data points (δ^13^C = -22.1 to -18.5‰) represents elephants that fed on a mixture of both C_3_ and C_4_ vegetation, in mosaic habitats of forest, woodland, and grassland. The third cluster, consisting of three outlying points to the right of the graph (δ^13^C = -13‰) are set apart by their high δ^13^C values, and represent elephants which consumed a substantial amount of C_4_ grass. Two elephants in this cluster with particularly high δ^15^N values (15.8 and 17.6‰) are from Arda-Arto, an area in central Ethiopia near what is today the Awash National Park. This area is dominated by dry bushland and grassland with wooded areas along the Awash River [97]. Powell-Cotton wrote in his hunting account of these two elephants that ‘…the next day’s march took us into a lovely country of low, well-wooded hills, with plenty of grass, which extended to Arda-Arto, on the bank of the Hawash’ with a further day’s journey leading them to ‘a sandy open plain, sparsely dotted with thorn trees’ [103]. Based on their isotope values, these elephants were likely feeding on the dry grasslands found in the more arid Afar region near Awash National Park. The third elephant in this group is from Rhino Camp in the northwest corner of Uganda, and the δ^15^N value is much lower than the samples from Ethiopia. This lower δ^15^N value could be a reflection of a more humid habitat or the consumption of a different type of C_4_ vegetation, as this habitat is near a permanent water source (Nile River) and historically received an average rainfall of over 1000 mm per year [99,100].

The δ^13^C and δ^18^O values measured in the carbonate of modern elephants in Fig 2 (A2) range from -14.6 to -5.8‰ and 24.3 to 33.3‰ respectively. Elephants from more arid locations (red symbols) have higher δ^18^O values (n = 5, mean = 32.4‰, stdev = 0.8‰) than samples from forest and high altitude environments (n = 2, mean = 26.2‰, stdev = 2.6‰). For example, one of these samples is from the Mau Escarpment, Kenya. Ambrose and DeNiro [104] surveyed a gradient of C_3_ to C_4_ plants along the Mau Escarpment and found that C_4_ plants were restricted to low altitudes with a sharp transition to C_3_ plants in higher altitudes. This elephant has a δ^13^C value indicative of C_3_ consumption, and low δ^18^O value, reflecting the high altitude of the habitat in which the elephant was likely feeding.

The δ^13^C and δ^18^O values measured in the carbonate of historic elephants in Fig 2 (B2) range from -16.8 to -7.6‰ and 22.6 to 32.2‰ respectively. A correlation between low δ^13^C and low δ^18^O values seen here indicates that elephants which primarily consumed C_3_ vegetation (lower δ^13^C values) lived in more humid and/or high altitude environments (lower δ^18^O values). There is also a similar trend found in the modern samples from more arid locations (red symbols) which have higher δ^18^O values (n = 3, mean = 31.4‰, stdev = 0.9‰) than the elephants living in forested/mountain locations (green symbols) (n = 6, mean = 27.4‰, stdev = 1.8‰).

The range of ^87^Sr/^86^Sr values (0.705 to 0.720) measured in all the modern elephant samples is wide (Fig 2, A3), although animals from the same reserve have a relatively conservative range of values. As expected, the elephant from Arusha (Rift Valley volcanics) has the lowest ^87^Sr/^86^Sr value in the data set (0.70512), though many samples fall within this lower ^87^Sr/^86^Sr range, including all of the Kenyan elephants (Garissa, Tana River, Mau Escarpment), reflecting the young geology of the Rift. The Saadani and Mikumi samples from near the Tanzanian coast reflect expected ^87^Sr/^86^Sr values typical of coastal sedimentary geology (0.71353 ± 0.002).

The ^87^Sr/^86^Sr values (0.705 to 0.736) for the historic elephants shown in Fig 2 (B3) exhibit a clear separation between those animals which roamed on younger (below 0.708) and older geology (above 0.710). Using a second isotope system (δ^13^C_collagen_), it is possible to further discriminate those elephants that consumed C_3_ vegetation in forests (δ^13^C less than -23‰) as these populations also have higher ^87^Sr/^86^Sr values (n = 3, mean = 0.726, stdev = 0.008) due to the older basement geology in the interior regions of East Africa.

## Discussion

### Trends in Provenanced Data Sets

For each of the isotopes measured, a distribution map is displayed in Figs 3–6 showing the isotopic variability of elephants across different habitats and relating those values to the distribution of vegetation cover, annual precipitation, elevation, and bedrock geology.

The variation in δ^13^C_collagen_ values in the modern and historic provenanced elephants is shown in Fig 3, with values ranging from -27.8‰ to -12.7‰, on a base map of vegetation cover. The elephants living in forests further inland have the most depleted δ^13^C values (darkest green symbols) and differ significantly from elephants from arid or mosaic habitats (Mann-Whitney Z-value for forest vs mosaic is 3.11 and for forest vs arid is -3.20, p<0.001 in each case). However, δ^13^C values for some elephants near the Great Lakes region indicate a substantial amount of C_4_ grass in the diet. From the vegetation map (Fig 3) it is possible to see that there are large areas of grassland along the lake shores, as well as areas such as the Queen Elizabeth National Park, Uganda, which has a mixture of short grassland, thicket and tall grassland [97,105]. Conversely, the strip of coastal forest along the Kenya and Tanzania coastline is reflected in the more negative δ^13^C value of a sample from Saadani National Park. Elephants from the area of savanna just to the west of this coastal belt have more mixed δ^13^C values reflecting the bushland and thicket in the habitat (light brown and yellow symbols).

The variation of δ^15^N values in elephants across the region ranges widely from 3.4‰ to 17.6‰, as shown against regional variations in annual precipitation in Fig 4. High δ^15^N values are measured in populations in the north-east of the region and within the more arid habitats of the Rift. As the rainfall pattern becomes higher farther inland and in clusters along mountain ranges, lower δ^15^N values are observed. As a result, elephants from arid locations have significantly different δ^15^N values than those from mountain/forest locations (Mann-Whitney Z-value for arid vs forest is -3.92, p<0.001). For both the historic and modern data sets, there was a significant negative correlation (r = -0.684, -0.538, p<0.005 respectively) between the annual precipitation and δ^15^N value of the elephant from each location. However, these correlations are reported with caution, as rain forest elephants can have higher δ^15^N values than those living in mixed grassland/woodland environments with lower rainfall due to the moisture availability in the soil and ‘openness’ of the nitrogen system in rainforest environments [67,68]. Thus it is important to note that high δ^15^N values are only consistently measured in populations living in regions of more extreme aridity.

The variation in δ^18^O values in the elephants is displayed on a base map of elevation in Fig 5, with values ranging from 22.6‰ to 33.3‰. For the provenanced data set, there is a significant positive correlation (r = 0.542, p<0.001) between δ^18^O values and longitude, or in other words, decreasing δ^18^O value with distance from the Indian Ocean coastline. The rainout effect is enhanced by continental topography since the mountains and tropical forests are in the interior, west of the Great Lakes. The modern and historic elephants which have high δ^15^N values (Garissa, Lugard Falls and Tana River, Arda-Arto) also have high δ^18^O values, as expected given that these locations are in low rainfall, lower altitude environments. The modern and historic elephants from arid locations have significantly different δ^18^O values than those from mosaic or forested environments (Mann-Whitney Z-value for arid vs mosaic is -3.20 and for arid vs forest is -4.09, p<0.001 in each case).

The variation in ^87^Sr/^86^Sr in the samples is displayed on a base map of bedrock geology in Fig 6 and shows the large geological variation across this region with ^87^Sr/^86^Sr values between 0.70512 and 0.73639. There is also a significant negative correlation (r = -0.698, p<0.001) between longitude and ^87^Sr/^86^Sr values in the provenanced data set. This negative correlation is primarily driven by the differentiation of strontium isotope values between the volcanic Rift Valley, with its comparatively young surface geology and thus low ^87^Sr/^86^Sr values, and the regions farther into the interior with much older surface geology creating comparatively higher ^87^Sr/^86^Sr values.

The results demonstrate that isotope data differentiate broadly between ivory obtained from elephants that routinely occupied different habitats and geographical areas across eastern Africa. For example, those elephants that lived in closed canopy forests in the interior of the region are characterised by their low δ^13^C values, low δ^18^O values, and high ^87^Sr/^86^Sr values. This combination of distinct isotope values is related both to the forested habitat and the older basement compared to the younger Rift volcanics to the east. Elephants roaming over Rift volcanics have low ^87^Sr/^86^Sr but a wide range of δ^13^C values due to the variety of vegetation types of the Rift. Coastal elephants reflect a sedimentary bedrock range in their ^87^Sr/^86^Sr values. Those which have rather low δ^13^C values inhabited coastal forests, whereas those inhabiting coastal savannas have higher δ^13^C values.

These isotope results therefore reflect not only the mosaic vegetation cover of East African elephant habitats, but also elephant migration to different patches of vegetation based on what is palatable and available in different seasons [43]. Codron et al. [43] concluded through studying elephant diet in Kruger Park over decades that elephants are ‘dietary generalists,’ and that there is considerable diversity at the individual level in terms of the amount of graze to browse consumed. This is represented in our data set by elephant tissue samples from Mikumi National Park, Tanzania, which have a large range of isotope values. Mikumi is a diverse landscape and forms a wildlife corridor with the Selous Game Reserve, so the variability measured in these elephants reflects their diverse and expansive habitat range. In the historic data set, two elephants from Wadelai, Uganda that were shot by Powell-Cotton in the same year have disparate δ^13^C_collagen_ values (-23‰ and -18‰) and ^87^Sr/^86^Sr values (0.720 and 0.716), but more similar δ^18^O values (25.9‰ and 27.4‰) (Fig 2, B1-3). These values reflect the vegetation in this region which includes forest, woodland, but also open grassland near Lake Albert and the tributaries that feed into it [97]. Powell-Cotton wrote that near Wadelai he ‘came upon a solitary elephant drinking from a little pool on an open expanse of grass’ [106] and in his diary for the day that he shot the elephant, he describes swampy expanses that elephants visited to drink [107]. Furthermore, this area straddles younger volcanics found in the westernmost part of the Rift and older basement at the edge of the Congo basin which would cause the differences in the ^87^Sr/^86^Sr values of these elephants [108]. Another factor here, as mentioned previously, is that male elephants have feeding patterns that are more versatile and less conservative than females and most of the elephants collected in the historic data set were from male elephants.

Overall, the results demonstrate patterns in both the historic and modern data sets for East African elephants that are useful for determining the origin of unknown ivory samples. δ^13^C values are low on the eastern coast due to coastal forest habitats and are more positive until the Lakes, mountains, and forests are reached further inland, where the values are lower. The lowest δ^13^C values are recorded in elephants living in closed canopy forested habitats in the interior. δ^15^N values are variable, but highest in elephants from arid habitats, with δ^18^O values following this trend. δ^18^O values also are lower in elephants which lived further inland and in higher altitude habitats. And finally, ^87^Sr/^86^Sr values are lowest in the Rift and higher going west towards the basement geology of the interior region. Thus, it should be possible using all four isotopes to better pinpoint the origins of historic elephants, with the proviso that vegetation and rainfall patterns have remained similar in these same habitats in the past.

### Historic Unprovenanced Ivory

**Fig 7:**
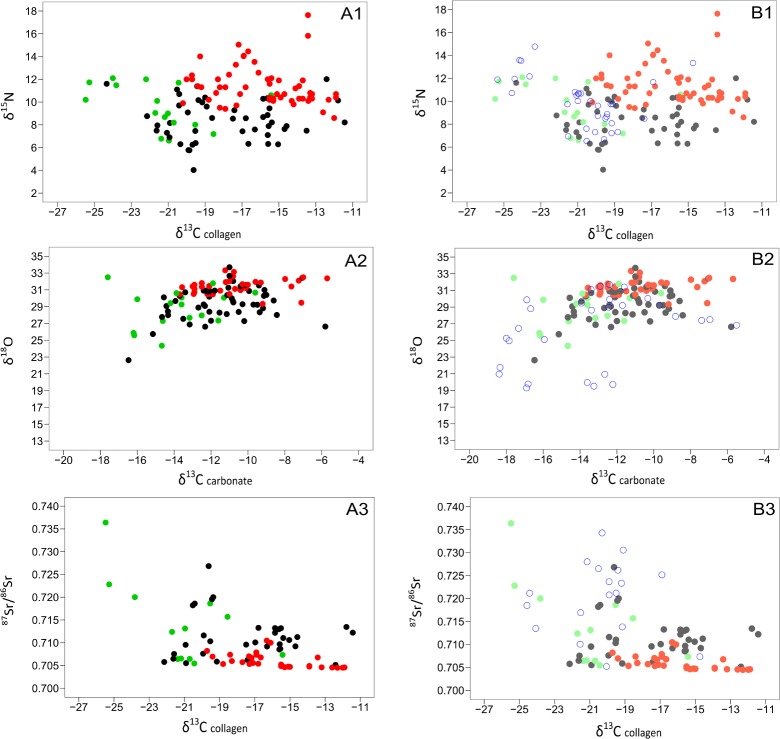
δ^13^C, δ^15^N, δ^18^O and ^87^Sr/^86^Sr values for modern, historic, and unprovenanced elephant samples from East Africa. (A1-3) Three plots of modern (post-1950) and historic elephant tissue samples collected from museum specimens, national parks in Tanzania (see S1 Table), and including modern published data from [39,41,42]. Colours of all samples correspond to habitat following [41] for East African elephant habitats: green is forest/mountain, black is savanna mosaic (incorporates woodland/bushland/grassland habitats) and red is arid (incorporates arid bushland and grassland habitats), with habitat descriptions following [97]. (A1) δ^13^C and δ^15^N values of collagen, (A2) δ^13^C and δ^18^O values of carbonate, and (A3) δ^13^C and ^87^Sr/^86^Sr values, from collagen and carbonate respectively. (B1-3) Three plots of all samples included in (A1-3) are lightly shaded and in blue open circles, the unprovenanced ivory samples from museum collections (see S1 Table). (B1) δ^13^C and δ^15^N values of collagen, (B2) δ^13^C and δ^18^O values of carbonate, and (B3) δ^13^C and ^87^Sr/^86^Sr values, from collagen and carbonate respectively.

Having explored trends in the provenanced data set, the results of the analyses of unprovenanced ivory (i.e. the piano keys, cutlery handles, and other ivory objects traded from East Africa) can be considered. In Fig 7 (A1-3), the results of the modern, historic, and additional published data [39,41,42] are coloured according to habitat zone as before (green = forest/mountain, black = mosaic, red = arid) and in Fig 7 (B1-3), the values from the modern and historic provenanced elephants are plotted in closed circles as in Fig 7 (A1-3), but with the addition of the unprovenanced ivory plotted in blue open circles. The majority of these ivory samples, including piano keys and cutlery handles, have δ^13^C values lower than -18‰, suggesting that they derived from elephants consuming substantial amounts of C_3_ vegetation (Fig 7, B1). Seven of the unprovenanced ivory samples plot with elephants from closed canopy forest habitats (δ^13^C values of -24‰ +/- 0.6). Only three of the unprovenanced samples have values higher than -18‰; they include a tusk from the Zanzibar museum and piano keys. Although all the piano keys from one piano were manufactured in Ivoryton, USA, one of the keys plots separately from the others primarily due to its low δ^13^C value of -16‰, with the others from the group with values averaging -20‰. Historical sources on piano key manufacturing in Ivoryton indicate that efforts were made to ensure uniform colour and texture across the keyboard and hence key tops were usually cut from the same tusk [109]. In this case, however, the ivory may be from a different elephant.

Fig 7 (B2) shows δ^18^O and δ^13^C from unprovenanced ivory in open blue circles. A large proportion of ruler blanks (blank ivory pieces that would have been made into rulers) have low δ^18^O and δ^13^C values very similar to samples from the Ituri Forest, Congo. Interestingly the δ^18^O values of many of the other blanks are outside the range of the provenanced samples, with lower δ^18^O values (less than -23‰) than the provenanced elephants from forested habitats. This could indicate that they are from other inland forests with a hydrological regime different from that of the provenanced and published data sets. Measured δ^18^O values in modern Central African elephants and mountain/forest elephants of East Africa are around 25‰ [41]. These unprovenanced samples may originate from higher altitude (low δ^18^O values), rainforest elephants farther west towards the Congo basin and within the distribution of West African monsoonal rainfall originating from the Atlantic rather than the Indian Ocean, as the source of the rainfall affects the oxygen isotope value of the drinking water. Alternatively, they might be derived from elephants that occupied mountainous habitats in Uganda/Rwanda where elephants are unknown today. Most of the other piano keys plot within the δ^18^O range of the East African elephants that consumed mixed vegetation in mosaic habitats of woodland and grassland.

Finally, the ^87^Sr/^86^Sr values for the unprovenanced samples are plotted with δ^13^C in Fig 7 (B3). Most of the ruler blanks and piano keys have high ^87^Sr/^86^Sr values (<0.720), plotting more closely to the provenanced samples from forested habitats and older geological ranges, such as those located farther into the East African interior. However, the δ^13^C results do not suggest that all of the ivory used for these piano keys was derived from closed canopy forest elephants, but rather that some were mixed C_3_ and C_4_ consumers, suggesting that they occupied different habitats. Thus, it is possible these ivories came from elephants that inhabited more open habitats but on older geology: the provenanced samples that are similar to these values are from Ruaha National Park, Tanzania and Uganda. The Korogwe archaeological elephant bone is an outlier compared to the other unprovenanced samples, as it has a low ^87^Sr/^86^Sr value, meaning that this elephant could have been local to the Pangani basin, as Korogwe and areas west towards Arusha and Kilimanjaro are located on a geological belt of young basalts.

**Fig 8:**
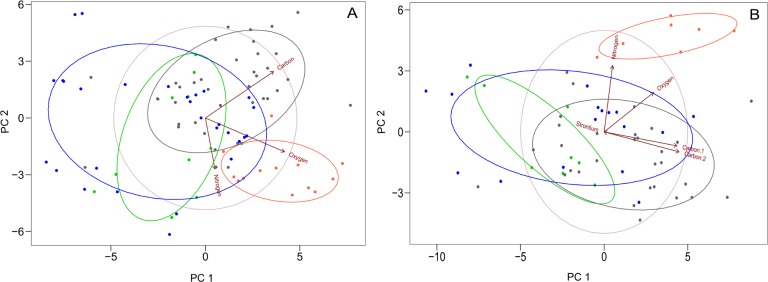
Principal component analysis plots. Colours of symbols and 95% confidence circles correspond to colours in previous figures, green is forest/mountain, black is savanna mosaic (incorporates woodland/bushland/grassland habitats), red is arid (incorporates arid bushland and grassland habitats), and blue is unprovenanced ivory. (A) represents the scores for the samples along PC 1 versus PC 2 with three of the isotopes included as variables (δ^13^C, δ^15^N, δ^18^O) given that more samples had data for these three isotopes than all four isotopes together. PC 1 explained 52.4% of the variance in the data set whilst PC 2 explained 28.1%. (B) represents the scores for the samples along PC 1 versus PC 2 with all of the isotopes included as variables (δ^13^C, δ^15^N, δ^18^O and ^87^Sr/^86^Sr). PC 1 explained 71.7% of the variance in the data set whilst PC 2 explained 19.5%.

In order to compare all of the isotopes together from the modern, historic, and published data sets, a principal component analysis was carried out. When three of the isotopes are compared (δ^13^C, δ^15^N, and δ^18^O), the first two principal components (Fig 8A) account for 80% of the total variance. When all the isotopes are compared (δ^13^C, δ^15^N, δ^18^O, and ^87^Sr/^86^Sr), the first two principal components (Fig 8B) account for 90% of the total variance. We analysed both because more samples had δ^13^C, δ^15^N, and δ^18^O values than ^87^Sr/^86^Sr values, so we could include more of the data in the analysis with three isotopes. The lines with arrows on the PCA plots visually represent how each of the isotopes affects the spread of the data along the principal component axes. Because each isotope pulls the data set in different directions, this demonstrates the importance of using all of the isotopes to more effectively separate the data set, which was also reported for isotope sourcing of modern African ivory samples [47]. Samples that plot in close proximity are related by the close range of their multiple isotope values and thus these elephants likely lived in a similar habitat.

Both of the plots consistently show that more of the unprovenanced samples match values measured in elephants from forest/mountain and mosaic habitats than arid ones (red symbols). The unprovenanced samples are also scattered quite widely, though there are a few clusters worth highlighting. Seven of the piano keys/ruler blanks have δ^13^C_collagen_ values lower than -23‰, and therefore cluster with the group of historic samples from closed canopy forest habitats. It is likely that these samples thus originated from the East African interior where these habitats exist, as there are no δ^13^C_collagen_ values this low from elephants elsewhere in East Africa along the coast or in the Rift. Another large group of ten unprovenanced ivory samples have high ^87^Sr/^86^Sr values, but with higher δ^18^O values and high δ^13^C values they are more similar to the values from the modern samples from Ruaha National Park, Tanzania, the published elephant value from Kasungu, Malawi [39], and the historic sample from Obbo, Uganda. A further two samples are set apart due to their low ^87^Sr/^86^Sr values, one from the archaeological site of Korogwe that is likely local to the area, and the other a ruler blank with an ^87^Sr/^86^Sr value of 0.705 and a δ^13^C_collagen_ value of -20‰. This ivory was likely from an elephant that inhabited forest or woodland on relatively young geology in the Rift such as around Mount Meru, Kenya.

Overall, the trends in the data sets highlight a number of issues regarding the provenancing of ivory samples from East Africa. The region is incredibly diverse in terms of its geology, vegetation, and climate, and therefore there are unique regions that provide ‘outlier’ numbers for certain isotope signatures, such as the Rift with its low ^87^Sr/^86^Sr values and the interior with its closed canopy rainforests and low δ^13^C values. We know from accompanying documentary sources that all of the unprovenanced pieces tested are from ivory obtained post-1890, in other words, after the hypothesised expansion of the ivory trade into the interior [7,8,20]. Many of the unprovenanced pieces did not come from arid environments, some came from closed canopy forests, and others likely from the Rift. Thus, it is possible to say that these samples likely did not originate from the narrow coastal strip along the southern Kenyan and northern Tanzanian coast where elephants are thought to have been eradicated by the 1890’s (see map in [20]). A portion of our unprovenanced ivory therefore supports the archival history, yet the remainder were from habitats that do not match our provenanced data set.

## Conclusion

Our study underlines the importance of a ‘multi-isotope’ approach for characterising East African habitats, as we demonstrated that all of the isotopes were necessary for explaining variation in the data set, and that without all of the isotope measurements, the ability to predict the habitat of origin of an unprovenanced sample declined substantially. A recent preliminary study of post-medieval ivory found in Amsterdam [110] reached a similar conclusion.

Despite the use of multiple isotopes, there are limitations of this technique, and in principle it may ultimately be more useful for determining where an elephant does *not* originate than pinpointing where it *does*. Assigning origin to elephant ivory becomes increasingly difficult the larger the scale, so using the technique on a continental scale, as has been a suggested application for modern illegal ivory confiscations [47], would not be possible without the addition of another method. For example, mitochondrial and nuclear DNA have been utilised recently to determine the likely source region of modern ivory confiscated from poachers and their middlemen (e.g. [111–114]). As African elephant genome databases continue to grow and sampling depth increases, it may be possible to use genetic markers to obtain more precise information regarding the location where the ivory was first obtained. More precise location information is particularly useful in terms of the modern trade, as better funding can be channelled to those regions and national parks in Africa which are witnessing increasing numbers of poached elephants. In terms of the historic ivory trade, better geographic provenancing of ivory could inform which habitats in Africa were most depleted of elephants by the ivory trade through times when there was a surge in extraction across the continent, such as the late 19^th^ century. This information might also be able to reveal trade connections between specific regions in Africa and the Middle East, Asia, and Europe from the early medieval period onwards by being able to provenance archaeological ivories traded from Africa across the globe. However, other methods may still be effective and can provide *additional* important information about ecological conditions and animal behaviour, as we report here with reference to the development of an isotopic approach to provenance historic ivory obtained from East Africa. Furthermore, the isotopic data sets created by this study and others could also be used on a smaller scale to understand elephant movement across park boundaries, as well as changes in the dietary ecology of these animals throughout time for the management of these landscapes into the future.

Ivory, whether from mammoth, Asian or African elephants, walrus, hippopotamus, narwhal or others, has been a desirable material among different human populations throughout the globe for millennia [115]. In this paper, we explored the geographical origins of ivory that was traded as part of the 19^th^ century East African caravan trade to understand the interactions between humans and elephants during a time when there was an exponential demand for ivory from this region of Africa. These results suggested that a range of habitats were exploited for elephants from the late 19^th^ to the mid-20^th^ centuries, but particularly interior regions of East Africa. Tracing the geographical origins of ivory, whether to address historical and archaeological questions or in the context of contemporary fears over the impact of poaching [116,117], is thus a necessary starting point for exploring the wide range of events and processes within which ivory was exploited. The practical task of doing so, however, depends on the robustness of the baseline data set and general knowledge of local ecology and habitats in the region to be explored.

## Supporting Information

S1 Table Isotope and location data for all samples. δ^18^O values are relative to VSMOW and the δ^13^C values of modern elephants have been corrected for depletion of ^13^C in atmospheric CO_2_ since the Industrial Revolution, due to burning of fossil fuels, for comparison with historic samples [90].
(XLSX)
